# Draft Genome Sequence of *Leptolyngbya* sp. KIOST-1, a Filamentous Cyanobacterium with Biotechnological Potential for Alimentary Purposes

**DOI:** 10.1128/genomeA.00984-16

**Published:** 2016-09-15

**Authors:** Ji Hyung Kim, Do-Hyung Kang

**Affiliations:** a Research Center for Viral Infectious Disease and Control, Korea Research Institute of Bioscience and Biotechnology, Daejeon, Republic of Korea; b Jeju International Marine Science Center for Research & Education, Korea Institute of Ocean Science & Technology, Jeju, Republic of Korea

## Abstract

Here, we report the draft genome of cyanobacterium *Leptolyngbya* sp. KIOST-1 isolated from a microalgal culture pond in South Korea. The genome consists of 13 contigs containing 6,320,172 bp, and a total of 5,327 coding sequences were predicted. This genomic information will allow further exploitation of its biotechnological potential for alimentary purposes.

## GENOME ANNOUNCEMENT

Cyanobacteria are photosynthetic prokaryotes, and hold promise as producers of sustainable bioresources for foods and other valuable products including bioenergy (1–3). However, selection of candidate strains for biotechnological applications has been a continuing challenge due to potential toxicity and low productivity. The genus *Leptolyngbya* which classified in the order *Pseudanabaenales* is filamentous cyanobacteria with a straight trichome, and known to be originated from various environments including extreme conditions (4). Therefore, several *Leptolyngbya* strains have recently been sequenced to understand its eco-physiological diversity (5–7). Recently, we reported on *Leptolyngbya* sp. KIOST-1 that possessed a number of advantageous characteristics for alimentary purposes with efficient productivity, high protein content, and lack of potential cytotoxicity (8). Here, we present its genome to exploit its biotechnological potential for industrial biomass production.

Genomic DNA was isolated using the DNeasy mini plant extraction kit (Qiagen) following the manufacturer’s protocols, and directly submitted for sequencing analysis. Genome sequencing was performed at Macrogen, Inc. (South Korea) using the Roche/454 pyrosequencing method on the Genome Sequencer FLX Titanium, and the obtained sequences were assembled using the GS De Novo Assembler software (v2.6). The resultant sequenced data consisted of a total of 366,508,458 bp and 560,014 reads with an average read length of 654.5 bp. Furthermore, the data included 548,755 assembled reads and 4,556 partially assembled reads. The *de novo* assembly resulted in eight scaffolds, and a total of 13 contigs that were longer than 500 bp were finally recovered after *in silico* finishing. The assembled draft genome was 6,320,172 bp, and its average G+C content was estimated to be 59.4%.

Genome annotation was acquired from the NCBI Prokaryotic Genome Annotation Pipeline (Bethesda, MD) and revealed 5,542 genes, 5,327 coding sequences, 162 pseudogenes, 7 rRNAs (4 for 5S, 2 for 16S, and 1 for 23S), 45 tRNAs, 1 noncoding RNA, and 26 frameshift genes. Additionally, gene ontology (GO) databases were used to functionally classify the predicted genes, and the results indicated that 23.6%, 25.5%, and 10.8% of the sequences included genes related to biological processes, molecular functions, and cellular components, respectively. However, more than 40% of the predicted genes failed to find a match in the GO database. In the GO category of biological processes, metabolic processes were the predominant subcategory, representing 38.1% of the genes. In the cellular component category, 20.7% of the genes were annotated as unknown, but 40.1% and 16.0% of the genes were associated with cell parts and membrane, respectively. Based on their molecular function, 44.9% of the genes were identified as being associated with catalytic activity.

Although no significant cytotoxicity was detected in our previous study (8), the entire genome of *Leptolyngbya* sp. KIOST-1 was scanned and no putative cyanotoxin genes were detected in it. This genomic information will contribute to better understanding for the genus *Leptolyngbya*, and allow further exploitation of its biotechnological potential for alimentary purposes.

### Accession number(s).

This whole-genome shotgun project has been deposited at DDBJ/EMBL/GenBank under accession no. JQFA00000000. The version described in this paper is version JQFA01000000.1.
